# Application of molecular imprinted polymer nanoparticles as a selective solid phase extraction for preconcentration and trace determination of 2,4-dichlorophenoxyacetic acid in the human urine and different water samples

**DOI:** 10.1186/s40201-014-0137-z

**Published:** 2014-11-14

**Authors:** Fariborz Omidi, Mohammad Behbahani, Hamid Sadeghi Abandansari, Alireza Sedighi, Seyed Jamaleddin Shahtaheri

**Affiliations:** Department of Occupational Health Engineering, School of Public Health, Shahroud University of Medical Sciences, Shahroud, Iran; Department of Chemistry, Shahid Beheshti University, Tehran, Iran; Department of Occupational Health, School of Public Health, Tehran University of Medical Sciences, Tehran, Iran; Department of Occupational Health Engineering, School of Public Health and Institute for Environmental Research, Tehran University of Medical Sciences, Tehran, Iran

**Keywords:** Molecular imprinted polymer nanoparticles, 2,4-dichlorophenoxyacetic acid, Selective preconcentration, Urine and water samples

## Abstract

A molecular-imprinted polymer nanoparticles (MIP-NP) for the selective preconcentration of 2,4-dichlorophenoxyacetic acid (2,4-D) is described. It was obtained by precipitation polymerization from methacrylic acid (the functional monomer), ethylene glycol dimethacrylate (the cross-linker), 2,2′-azobisisobutyronitrile (the initiator) and 2,4-D (the template molecule) in acetonitrile solution. The MIP-NPs were characterized by thermogravimetric analysis, and by scanning electron microscopy. Imprinted 2,4-D molecules were removed from the polymeric structure using acetic acid in methanol (15:85 v/v %) as the eluting solvent. The sorption and desorption process occur within 10 min and 15 min, respectively. The maximum sorbent capacity of the molecular imprinted polymer is 89.2 mg g^−1^. The relative standard deviation and limit of detection for water samples by introduced selective solid phase extraction were 4.2% and 1.25 μg L^−1^, and these data for urine samples were 4.7% and 1.80 μg L^−1^, respectively. The method was applied to the determination of 2,4-D in the urine and different water samples.

## Introduction

In recent years, the hazards of using pesticides have been accentuated by the sharp rise in their use in agriculture and industry. Phenoxy herbicides compounds are currently among the most frequently used pesticides worldwide [1]. They have been used on a large scale in agriculture to control the growth of broad-leaved weeds on rice, maize, wheat, and in post-emergence applications in most developing countries [2,3]. Among them, 2,4-dichlorophenoxyacetic acid (2,4-D) is a common important phenoxy herbicide that is selective, systemic auxin-type herbicide extensively used throughout the world for the past 50 years [4]. Exposures to 2,4-D occurs not only in general population groups through trace residues in the diet, from drinking water and direct contact with lawns recently treated with 2,4-D [5] but also persons who occupationally handle (mix, load or apply) it. 2,4-D is rapidly absorbed into the body following ingestion, inhalation and/or dermal contact and is rapidly excreted, predominantly in the urine unchanged [6,7]. Some recent well-documented studies show an association between the agricultural use of the 2,4-dichlorophenoxyacetic acid (2,4-D) and the risk of non- Hodgkin’s lymphoma [8]. It has been classified as possibly carcinogenic to humans (IARC, group 2B) [4]. Furthermore, continuous use of 2,4-D may cause soil percolation and groundwater contamination [9]. Due to the presence of the carboxylate group in its structure, this compound can react with metal ions to form complexes which are sparingly soluble in water. The World Health Organization (WHO), U.S. Environmental Protection Agency (EPA), and national governments such as New Zealand and United State of America (USA) regulation set the maximum contaminant level (MCL) of chlorophenoxy acid herbicides in drinking water in the range of 10-70 ng mL^−1^ [10,11]. Because of its adverse effect on humans, sensitive determination of 2,4-D in environmental and biological samples is necessary.

In the literature, several methods have been developed for determining 2,4-D in different environmental samples, including solid phase microextraction gas chromatography-mass spectrometry (SPME-GC-MS) [12], solid phase extraction high-performance liquid chromatographic with UV detector (SPE-HPLC-UV) [13] and liquid - liquid extraction gas chromatography-mass spectrometry (LLE-GC-MASS) [14] methods. The mentioned methods have major drawbacks as they are very time consuming procedure, requiring expensive instrumentation, affected by matrix effects and have low selectivity and recoveries. According to the complexity of matrix and low content of 2,4-D in real samples, an efficient sample treatment technique with high clean-up and high enrichment factor are required prior to analysis. Thus, development of a rapid and selective extraction technique for determination of 2,4-D from environmental and biological matrices is of great importance.

In recent years, molecularly imprinted polymers (MIPs) and ion imprinted polymers (IIPs) have been extensively used (e.g. SPE sorbent) for preconcentration and high efficient separation of different trace analytes in complex matrices [15-28], based on their unique imprinting and recognition properties. MIPs demonstrate high potential for sample clean-up of very complicated matrix [29], solid-phase extraction [30-34], and solid-phase micro extraction fibers [35]. The most widely employed preparation method for synthesis of MIPs is bulk polymerization. The prepared polymer with this method is then ground and sieved, and particles of usually about 25 mm are collected for subsequent studies.

Although the bulk MIP prepared by conventional methods exhibits high selectivity [36,37], some disadvantages were suffered, such as the heterogeneous distribution of the binding sites, embedding of most binding sites, and poor site accessibility for template molecule [38]. Consequently, the consideration of researchers has moved toward achieving highly uniform spherical imprinted particles, particularly on the nanoscale [39]. In contrast to monoliths, MIPs possess cavities designed for a target analyte, providing a retention mechanism based on molecular recognition; by this, imprinted cavities are more easily available to templates and the binding kinetic is increased [40]. Different methods are used to obtain MIPs Nano particles, namely suspension, multistep swelling and precipitation polymerization. The different methods of preparation have been well documented in a recent review by Haginaka [41]. Among them, the precipitation technique is the most convenient one, because it is a homogeneous, one-step synthesis and does not require the use of a surfactant or stabilizer that can remain adsorbed to the surface of the polymer, interfering with the selective binding of the target to the imprinted materials. The first prepared MIPs, using precipitation polymerization technique, were reported by Ye et al. [42]. The polymer particles were obtained according to this procedure, have more uniform particles size and require no crushing and sieving steps in comparison tothe polymers obtained by bulk polymerization.

The aim of the present study was the preparation of 2,4-D imprinting polymer via non-covalent precipitation polymerization as well as its application in selective sample preparation in order to eliminate the matrix effects. HPLC-UV was used for determination of 2,4-D after preconcentration by MIP-NPs in urine and different water samples.

## Experimental

### Reagents and chemicals

2,4-D was purchased from Fluka (Buchs, Switzerland). Azobisisobutyronitrile (AIBN) as the initiator was obtained from Acros Organic (NJ, USA). Methacrylic acid (MAA), ethylene glycol dimethacrylare (EGDMA), acetic acid (HOAC), sodium hydroxide (NaOH), acetonitrile (ACN) and methanol (MeOH) were purchased from Merck (Darmstadt, Germany, www.merck.de). All the reagents used were of analytical grade. Ultrapure water was prepared using a Milli-Q® system (Millipore, Milford, MA, USA). Stock standard solution of 2,4-D (1000 mg L^−1^) was prepared in methanol and stored at refrigerator.

### Instrumentation

All measurements were performed by a reversed-phase HPLC system from the Knauer Company (Germany), consisting of a K-1001 series high-pressure pump, a K-2006 photo diode-array detector and a VS injection valve, equipped with a 20-μL loop. The analytes were separated on a Chromolith performance RR-C_18_ 100 mm × 4.6 mm i.d. (MerchKGa A, Germany) and column guards (Chromolith Guard Cartridge Kit RP- C_18_ and 5 cm × 4.6 mm i.d., 5 μm). The mobile phase composition was 79.5% acetonitrile, 19.5% purified water and 1% acetic acid The flow-rate was set at 1 mL min^−1^ in isocratic mode with the detector’s wavelength set at 280 nm. A digital pH meter, WTW Metrohm 827 Ion analyzer (Herisau, Switzerland), equipped with a combined glass calomel electrode was used for the pH adjustments at 25 ± 1°C temperature. Scanning electron microscopy (SEM) was carried out by gently distributing the powder sample on the stainless steel stubs, using SEM (Philips, XL-30, Almelo, the Netherlands) instrument. BAHR-Thermoanalyse GmbH (Germany) was used to determine the thermal properties of synthesized polymers with employing, heating and cooling rates of 10°C min^−1^ and using a condenser as the coolant. The samples were weighed as a thin film and carefully packed into a clean aluminum pan (11.5-12.5 mg), and sealed by crimping an aluminum lid on the pan (Shimadzu universal crimper). An Al_2_O_3_ empty pan sealed with a cover pan was used as a reference sample. A scanning range of 10 to 800°C was used for samples at 10°C min^−1^ in nitrogen gas.

### Synthesis of molecular imprinted and non imprinted polymer nanoparticles

**Figure 1:**
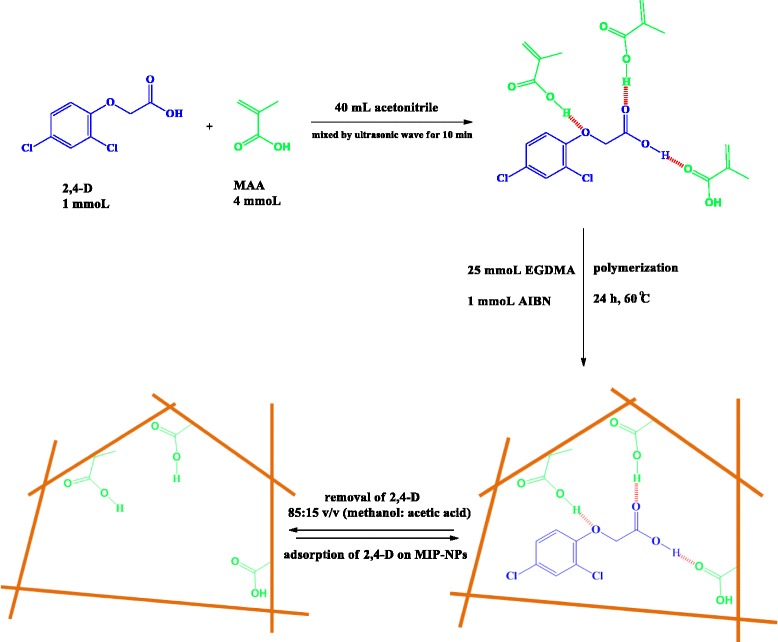
**A scheme for the synthesis of 2,4-D imprinted polymer nanoparticles.**

Precipitation polymerization technique was used for preparation of MIP-NPsaccording to following procedure (Figure 1): 1 mmol of 2,4-D (template) 4 mmol of MAA (functional monomer)which were dissolved in 40 mL of acetonitrile (progen solvent)in a 100 mL glass flask. The solution was mixed by ultrasonic wave for 10 min and then 1 mmol of AIBN (initiator) and 25 mmol of EGDMA (cross-linker agent) were added to the solution. The polymerization mixture was purged with a gentle flow of nitrogen gas for 6 min and the flask was sealed under nitrogen then; polymerization was performed in an oil bath at 60°C for 24 h in the presence of nitrogen under magnetic stirring at 400 rpm. The polymer nanoparticles were collected by centrifugation and washed with acetone and methanol, respectively for removing additional reagents and solvent. The template was removed from the cavity of MIP-NP using soxhlet extraction (acetic acid-methanol (85:15 v/v)), This step was confirmed through analysis of the eluted template molecules by HPLC analysis. Afterwards, the polymer was washed with deionized water until neutralization was obtained. Furthermore, non-imprinted polymers (NIP), as a reference, was prepared using the same process without the addition of the template molecule.

### Preparation of standard solutions and real samples

Stock standard solution of the 2,4-D was prepared at concentration level of 1000 mg L^−1^ in methanol. The stock solution was protected from light and stored at 4°C and brought to ambient temperature just prior to use. The required working solutions were prepared daily in double-distilled water prior to use. For analysis of urine sample, 2 mL of spiked urine was diluted to 7.0 mL with ultrapure water. The water samples, including distilled water, tap water (Tehran, Iran), river waters obtained from Siahrood river and Derka river (Ghaemshahr, Iran) and sea water (Caspian Sea, Sari, Iran) were collected in cleaned polyethylene bottles and filtered through a 0.45 mm pore size nylon filter (Millipore) immediately after sampling. The amounts of 2,4-D was successfully determined by the present method.

### Extraction procedure

**Figure 2:**
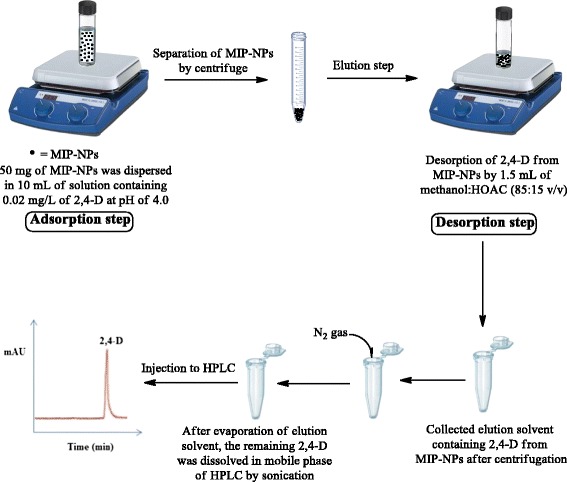
**A schematic illustration of extraction procedure.**

All factors affecting sorption and desorption steps were investigated using batch method. Extraction of 2,4-D molecules from modeling solutions and real samples is followed by two steps: sorption and desorption. In the sorption step, the pH of sample solution was adjusted to 4.0 by drop wise addition of 2 mol L^−1^ sodium hydroxide or hydrochloric acid solutions. Then, 50 mg of dried polymer was suspended in aqueous solution (10 mL) containing 0.02 mg L^−1^ concentration of 2,4-D and stirred for 10 min with a magnetic stirrer, after that the polymer nanoparticles were separated from the solution by centrifugation. In washing step, in order to remove the potential interfering compounds and loosely retained molecules, the MIP-NPs were washed several times with double distilled water, then in desorption step, the elution of 2,4-D from MIP-NPs was performed by 1.5 mL of HOAC in methanol (15:85 v/v %) for 15 min. The eluate (separated by centrifuge) evaporated to almost dry under a gentle stream of nitrogen and the residue was dissolved in 0.3 mL mobile phase under sonication. Finally, 20 μL of the separated phase was drawn out by a Hamilton syringe and directly injected into the HPLC-Uv for analysis. Figure 2 provides a scheme for illustration of the mentioned extraction procedure.

Extraction percent of 2,4-D was calculated by the following equation:

$$ \mathrm{Adsorption}\ \mathrm{efficiency}\%={\mathrm{C}}_{\mathrm{A}}-{\mathrm{C}}_{\mathrm{B}}/{\mathrm{C}}_{\mathrm{A}}\times 100 $$

C_A_ and C_B_ are the concentrations of 2,4-D before and after extraction in the solution, respectively.

## Results and discussion

### Characterization of the synthesized molecular imprinted polymer nanoparticles

**Figure 3:**
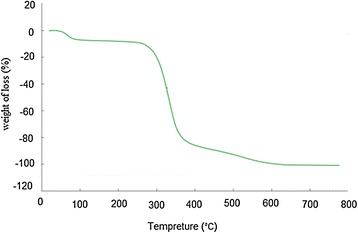
**The thermogram of synthesized molecular imprinted polymer nanoparticles.**

**Figure 4:**
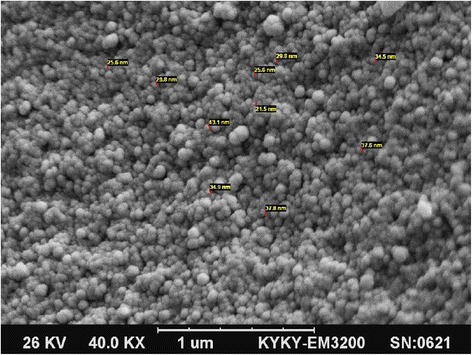
**The SEM micrograph of synthesized molecular imprinted polymer nanoparticles.**

The resulting nanosized imprinted polymers were characterized by scanning electron microscopy (SEM), and thermogravimetric analysis (TGA). Thermal stability of the synthesized imprinted polymer nanoparticles was evaluated by TGA. Figure 3 shows TGA plot for the imprinted polymer. TGA plot for MIP-NPs can prove the thermal stability of the synthesized polymer. The morphology of the MIP-NPs was assessed by scanning electron microscopy, and the SEM micrograph is shown in Figure 4. As you can see in the Figure 4, the particle size of synthesized imprinted polymer was in range of 25-37 nm. These observation indicate that the formation 2,4-D imprinted nano particles was performed successfully and it can be used as a nano-size selective solid phase for very fast extraction of ultra-trace amounts of 2,4-D. The prepared MIP in the nanosize particles have many advantages such as: fast extraction of analyte from the real samples, high adsorption capacity of the prepared nanosized MIP toward conventional MIP according to the simple diffusion of target molecules to formed cavity due to high surface area of nanosized MIP.

### Removal step

#### Effect of solution’s pH

**Figure 5:**
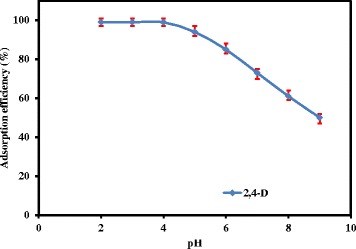
**The effect of solution’s pH on the adsorption efficiency of 2,4-D on MIP-NPs.** (Conditions: 2,4-D concentration: 0.02 mgL^−1^, Sample volume: 10 mL, Adsorption time: 10 min).

To evaluate the effect of pH on the extraction efficiency, the pH of 10 mL of sample solutions containing 0.02 mg L^−1^ of 2,4-D was adjusted in the range of 2-9. The pH of the suspensions was adjusted to desired values by adding sodium hydroxide or hydrochloric acid. Figure 5 illustrates the effect of solution’s pH on percent of Adsorption efficiency. As it shows, the adsorption of 2,4-D by MIP-NPs was approximately constant from pH 2.0 to 4.0 but decreased from pH 5.0 to 9.0. Therefore, the pH of 4.0 was chosen as optimized pH, for quantitative adsorption of 2,4-D on the synthesized MIP-NPs in the subsequent experiments.

#### Equilibrium sorption time

**Figure 6:**
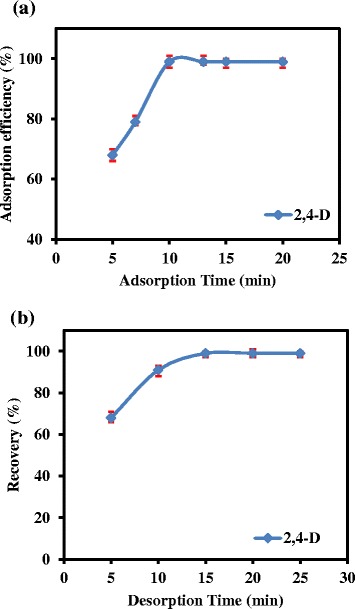
**The effect of adsorption time (a) and desorption time (b) on the adsorption efficiency and recovery of 2,4-D from MIP-NPs.**

In a typical uptake kinetics test, 50 mg of the sorbent was added to 10 mL of 0.02 mg L^−1^ 2,4-D aqueous solution at pH 4.0. The resulting suspension was stirred in different times (i.e., from 5 to 20 min) under magnetic stirring (Figure 6a). Consequently, an optimum equilibration time of 10 minutes was obtained for quantitative removal of 2,4-D from solution into the solid phase.

### Desorption step

#### Choice of eluent and desorption time

Complete removal of template from synthesized polymer isa very crucial step to guarantee the absence of memory effect. In this step, several solvents were examined; whose results are provided in Table 1. It is obvious that HOAC in methanol (15:85 v/v %) shows better recovery compared with the other solvents. Acetic acid can affect the hydrogen bonding between template and functional monomer, so that the removal of the template would be easier. In subsequent experiments (Table 2), 1.5 mL of HOAC in methanol (15:85 v/v %) was used as optimum eluent.

**Table 1:** **The effect of elution solvent type (The volume for each elution solvent was 5 mL) on the recovery of the target molecule from MIP-NPs (The obtained results are the mean of three measurements)**

**Elution solvent**	**Concentration (V/V (%))**	**R** ^**a**^ **± S** ^**b**^
Methanol	100	70.0 ± 1.4
Water	100	52.0 ± 1.6
Methanol: Acetic acid	95:5	82.0 ± 1.0
Water: Acetic acid	95:5	66.0 ± 1.2
Methanol: Acetic acid	90:10	90.0 ± 1.7
Methanol: Acetic acid	85:15	99.0 ± 1.0
Methanol: Acetic acid	80:20	97.0 ± 0.8
Methanol: Acetic acid	75:25	89.0 ± 1.0

^a^Recovery (%) ^b^Standard deviation.

**Table 2:** **The effect of elution solvent volume on the recovery of the target molecule from MIP-NPs (The obtained results are the mean of three measurements)**

**Elution solvent (V/V (%))**	**Volume (mL)**	**R** ^**a**^ **± S** ^**b**^
Methanol: Acetic acid (85:15)	5	99.0 ± 0.8
Methanol: Acetic acid (85:15)	4	99.0 ± 1.0
Methanol: Acetic acid (85:15)	3	99.0 ± 0.9
Methanol: Acetic acid (85:15)	2	99.0 ± 1.0
Methanol: Acetic acid (85:15)	1.5	99.0 ± 1.0
Methanol: Acetic acid (85:15)	1.0	74.0 ± 1.2

^a^Recovery (%) ^b^Standard deviation.

In order to investigate the optimum desorption time, various times were examined in the range of 5 to 25 min, while other parameters were kept in optimum conditions. According to measurements, extraction recovery was increased up to 15 min and it was constant in longer times. Therefore, 15 minutes can be considered to be the best quantitative time for the elution of 2,4-D from the imprinted polymer (Figure 6b).

### The effect of centrifugation time

The final parameter which evaluated and optimized was centrifugation time. If the centrifugation time is not enough, the MIP nanoparticles cannot be completely collected on bottom of the vial. A series of extraction with varying centrifugation times from 1 to 5 min at a rate of 3000 rpm were performed. The extraction recovery for the analytes was lower when the centrifugation time was shorter than 3 min. However; longer centrifugation has not significant effect on the extraction efficiency of the target molecule. Therefore, 3 min was selected as the optimum centrifuging time.

### Investigation of the selectivity of the synthesized molecular imprinted polymer for 2,4-D

The effect of interfering agents on extraction recovery of 2,4-D were investigated in the presence of methylbenzene, aniline and 2-(4-chloro-2-methylphenoxy) propanoic acid with hundred fold concentration as interfering agents separately, while the other parameters were considered to be constant. The extraction recovery of more than 95% was obtained for 2-4-D in the presence of potentially interfering agent because of the high selectivity of synthesized MIP-NPs that allows its potential application as a sample preparation and clean up technique for analysis of 2,4-D in different matrices in the presence of interfering agents.

### Comparison of MIP and NIP

In order to compare MIP and NIP, two extractions by both MIP and NIP in water sample were assessed under the optimal conditions. As it has shown (Table 3), because of the high capacity of MIP for extraction of 2,4-D in comparison with NIP, the extraction recovery of MIP is more than that of NIP. The obtained results can prove the successful engineering of cavity for 2,4-D in the structure of synthesized MIP-NPs. In order to evaluate the maximum adsorption capacity, the difference between concentration of the solution before extraction and the concentration of the solution after extraction was calculated. The sorption capacities of the MIP and NIP were calculated to be 89.2 mg g^−1^ and 12.1 mg g^−1^, respectively.

**Table 3:** **The comparison between extraction recovery and adsorption capacity of MIP and NIP**

**Sorbent**	**R** ^**a**^ **± S** ^**b**^	**Adsorption capacity (mg g** ^**−1**^ **)**
Molecular imprinted polymer (MIP)	99.0 ± 1.0	89.2
Non imprinted polymer (NIP)	34.0 ± 1.3	12.1

^a^Recovery (%) ^b^Standard deviation.

### The effect of interfering agent

The effect of interfering agents on the extraction recovery of 2,4-D were investigated in the presence of atrazine, 3,4-Dichlorophenol and 2,4,6-Trichlorophenol with tenfold concentration as interfering agents separately, while, the other parameters were considered to be constant. The extraction recovery of more than 95% was obtained for 2,4-D in the presence of potentially interfering agents because of the high selectivity of MIP that allows its potential application as a sample preparation tool for analysis of 2,4-D extracted from environmental and biological samples.

### Statistical and calibration parameters

Under optimized conditions that have been described, the MIP-NPs-SPE showed a linear calibration curve within concentration ranging from 5 to 800 μg L^−1^. The least square equations at above linear dynamic range for water and urine samples were as follows:

$$ \begin{array}{l}\mathrm{Peak}\ \mathrm{area}=0.949\ {\mathrm{C}}^{\mathrm{a}}\left(\upmu \mathrm{g}\ {\mathrm{L}}^{-1}\right)+0.579,{\left({\mathrm{R}}^2=0.99\right)}^{\mathrm{a}}\mathrm{Concentration}\ \mathrm{o}\mathrm{f}\ 2,4-\mathrm{D}\ \left(\mathrm{F}\mathrm{o}\mathrm{r}\ \mathrm{water}\right)\\ {}\mathrm{Peak}\ \mathrm{area}=0.939\ {\mathrm{C}}^{\mathrm{a}}\left(\upmu \mathrm{g}\ {\mathrm{L}}^{-1}\right)+0.655,{\left({\mathrm{R}}^2=0.99\right)}^{\mathrm{a}}\mathrm{Concentration}\ \mathrm{o}\mathrm{f}\ 2,4-\mathrm{D}\ \left(\mathrm{F}\mathrm{o}\mathrm{r}\ \mathrm{urine}\right)\end{array} $$

The limits of detections, defined as C_LOD_ =3S_b_/m, where S_b_ is the standard deviation of seven replicate blank signals and m is the slope of the linear section of calibration curve after preconcentration, for a sample volume of 10 mL, were found to be 1.25 μg L^−1^ and 1.80 μg L^−1^ for 2,4-D in water and urine samples, respectively. Also, the relative standard deviations for eight separate batch experiments with 50 mg of sorbent for determination of 0.1 μg 2,4-D in 10 mL of water were calculated to be 4.2% and 4.7% in water and urine samples, respectively.

### Determination of 2,4-D in different water and urine samples

In order to evaluate the analytical applicability of the proposed method, determination of 2,4-D in urine and different water samples such as distilled water, tap water (Tehran, Iran), river waters obtained from Siahrood river and Derka river (Ghaemshahr, Iran) and sea water (Caspian Sea, Sari, Iran) was carried out. Table 4 shows that the results of three replicate analyses of each real sample obtained by the method are in good agreement with the spiking amounts. The repeatability of the method was demonstrated by the mean relative standard deviation (R.S.D.). Also, the relative recovery is defined as following equation:

$$ \mathrm{R}\mathrm{R}=\left({\mathrm{C}}_{\mathrm{found}}\hbox{--} {\mathrm{C}}_{\mathrm{real}}/{\mathrm{C}}_{\mathrm{added}}\right)\times 100 $$

**Table 4:** **Determination of 2,4-D in urine and different water samples**

**Sample**	**Analyte**	**C**_**added**_	**C**_**founded**_	**RSD %**_**Intra-day**_ **(N = 3)**	**RSD %**_**Inter-day**_ **(N = 3)**	**Relative recovery (%)**
Distilled water	2,4-D	---	---	---	---	---
		50.0 μg L^−1^	49.3 μg L^−1^	3.1	3.6	98.6
Tap water	2,4-D	---	---	---	---	---
		50.0 μg L^−1^	49.2 μg L^−1^	3.4	3.7	98.4
Siahrood river water	2,4-D	---	---	---	---	---
		50.0 μg L^−1^	49.4 μg L^−1^	3.5	4.3	98.8
Derka river water	2,4-D	---	1.9 μg L^−1^	---	---	---
		50.0 μg L^−1^	51.4 μg L^−1^	4.1	4.4	99.0
Sea water	2,4-D	---	---	---	---	---
		50.0 μg L^−1^	49.1 μg L^−1^	4.2	4.5	98.2
Urine	2,4-D	---	---	---	---	---
		50.0 μg L^−1^	48.9 μg L^−1^	4.1	4.6	97.8

**Figure 7:**
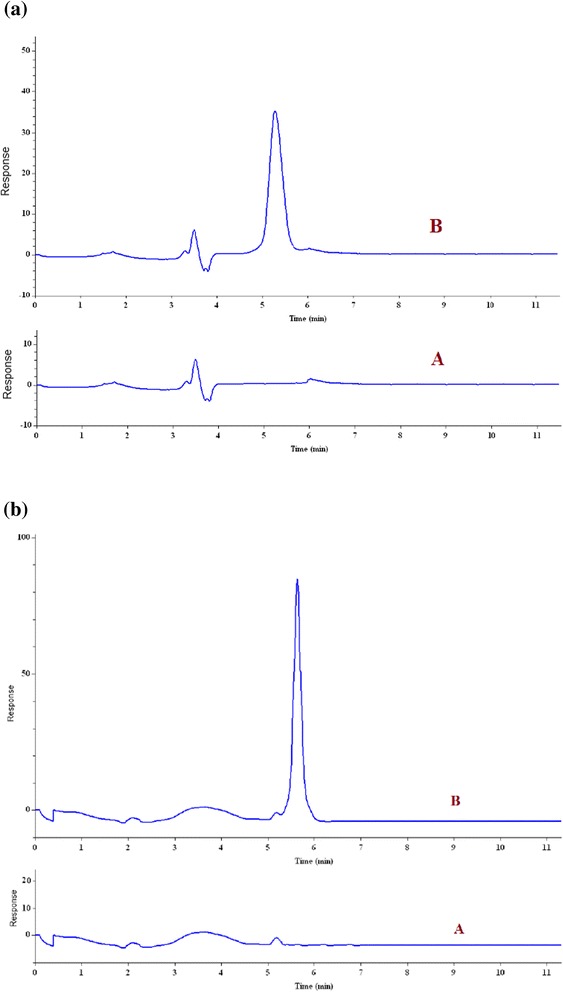
**The chromatograms of the (A) non-spiked and (B) spiked (100 μgL** ^**−1**^ **) sea water sample after extraction under optimum conditions (a) and the chromatograms of the (A) non-spiked and (B) spiked (100 μgL** ^**−1**^ **) urine sample after extraction under optimum conditions (b).**

Where C_found_, C_real_, and C_added_ are the concentrations of analyte after addition of known amount of standard in the real sample, the concentration of analyte in real sample and the concentration of known amount of standard which was spiked to the real sample, respectively. 2,4-D trace determination in water and urine samples, using MIP-NPs-SPE as a reliable sample treatment technique can be conducted. Figure 7 provides chromatograms for determination of 2,4-D in spiked urine and sea water samples.

## Conclusion

The molecular imprinted polymer is a selective sample preparation technique for preconcentration of molecules such as 2,4-D from aqueous solutions. The MIP-NPs has been synthesized by precipitation polymerization technique. The analytical characteristics such as good precision and preconcentration factor, high quantities of recoveries, wide dynamic linear range, and low detection of limit were achieved due to the powerful efficiency of the MIP-NPs-SPE method. Other advantages of suggested method are: lack of matrix effect, low consumption of organic solvent, environmentally friendly, safe, simplicity and selectivity. The synthesized MIP-NPs can be used repeatedly (7 times) with no significant decrease in its binding affinities. Due to relatively high preconcentration factor, trace amount of 2,4-D at μg L^−1^ levels can be determined and separated by MIP-NPs.
